# Optimization of a New Antioxidant Formulation Using a Simplex Lattice Mixture Design of *Apium graveolens* L., *Coriandrum sativum* L., and *Petroselinum crispum* M. Grown in Northern Morocco

**DOI:** 10.3390/plants12051175

**Published:** 2023-03-03

**Authors:** Ghizlane Nouioura, Meryem Tourabi, Asmae El Ghouizi, Mohammed Kara, Amine Assouguem, Asmaa Saleh, Omkulthom Al Kamaly, Faiçal El Ouadrhiri, Badiaa Lyoussi, El Houssine Derwich

**Affiliations:** 1 Laboratory of Natural Substances, Pharmacology, Environment, Modeling, Health and Quality of Life (SNAMOPEQ), Faculty of Sciences Dhar El-Mehraz, Sidi Mohamed Ben Abdellah University, Fez 30000, Morocco; 2 Laboratory of Biotechnology, Conservation and Valorisation of Natural Resources (LBCVNR), Department of Biology, Faculty of Science Dhar El Mahraz, Sidi Mohamed Ben Abdellah University, Fez 30000, Morocco; 3 Laboratory of Functional Ecology and Environment, Faculty of Sciences and Technology, Sidi Mohamed Ben Abdellah University, Imouzzer Street, Fez 30000, Morocco; 4 Department of Pharmaceutical Sciences, College of Pharmacy, Princess Nourah bint Abdulrahman University, Riyadh 11671, Saudi Arabia; 5 Laboratory of Engineering, Molecular Organometallic Materials and Environment, Faculty of Sciences, Sidi Mohamed Ben Abdellah University, Fez 30000, Morocco; 6 Unity of GC/MS and GC, City of Innovation, Sidi Mohamed Ben Abdellah University, Fez 30000, Morocco

**Keywords:** *P. crispum* M., *C. sativum* L., *A. graveolens* L., mixture design, antioxidant activity

## Abstract

A statistical Simplex Lattice Mixture design was applied to develop a new formulation based on a combination of three plants grown in northern Morocco: *Apium graveolens* L., *Coriandrum sativum* L., and *Petroselinum crispum* M. We examined the extraction yield, total polyphenol content (TPC), 2′2-diphenyl-l-picrylhydrazyl (DPPH) radical scavenging activity, and total antioxidant capacity (TAC). The results of this screening study showed that *C. sativum* L. had the highest content of DPPH (53.22%) and TAC (37.46 ± 0.29 mg Eq AA/g DW) compared to the other two plants, while *P. crispum* M. showed the highest TPC (18.52 ± 0.32 mg Eq GA/g DW). Furthermore, the ANOVA analysis of the mixture design showed that all three responses (DPPH, TAC, and TPC) were statistically significant, with determination coefficients of 97%, 93%, and 91%, respectively, and fit the cubic model. Moreover, the diagnostic plots showed good correlation between the experimental and predicted values. Therefore, the best combination obtained under optimal conditions (P1 = 0.611, P2 = 0.289, P3 = 0.100) was characterized by DPPH, TAC, and TPC of 56.21%, 72.74 mg Eq AA/g DW, and 21.98 mg Eq GA/g DW, respectively. The results of this study reinforce the view of stimulating the effect of plant combinations to achieve better antioxidant activities, thus providing a better formulation using designs of mixtures for the food industry and in cosmetic and pharmaceutical applications. Moreover, our findings support the traditional use of the Apiaceae plant species in managing many disorders cited in the Moroccan pharmacopeia.

## 1. Introduction

In recent decades, natural antioxidants have drawn increased interest [1]. Indeed, polyphenols are secondary metabolite compounds of plants and contain many combinations, including flavonoids, phenolic acids, and other compounds [2,3]. Moreover, a number of recent studies have focused on the biological properties of polyphenols and have revealed that these compounds protect human health against many diseases, especially those caused by oxidative stress, such as diabetes, cancer [4], chronic inflammation, and cardiovascular diseases [5], as well as afflictions caused by bacterial and viral agents [6].

The antioxidant, anti-inflammatory, antiproliferative, and anti-aging properties of natural polyphenols are closely related to their capacity to remove oxidative products such as reactive oxygen species (ROS) and reactive nitrogen species (RNS), which are highly reactive oxidized molecules that can destroy critical biological molecules such as proteins, lipids, carbohydrates, DNA, and RNA [7,8].

Natural phenolic compounds can suppress the gene expression of proinflammatory mediators such as cyclooxygenase (COX), as well as up- or downregulate transcriptional proinflammatory mediators such as nuclear factor-κB (NF-κB) or nuclear factor erythroid factor 2-related factor 2 (Nrf-2) [9]. Plant phenolic compounds can also act as inhibitors of many enzymes involved in several human diseases. For instance, they can inhibit the angiotensin-converting enzyme (ACE) in hypertension, acetylcholinesterase in Alzheimer’s disease, the carbohydrate hydrolyzing enzyme in type 2 diabetes mellitus, and tyrosinase in skin hyperpigmentation [10].

Besides their biological and beneficial health effects, phenolic compounds have aroused increasing interest in other fields, such as food industries (they provide flavor to food products) due to their nutritional value [11]. Natural plant antioxidants are also used in food conservation [12].

Parsley (*Petroselinum crispum* M.), coriander (*Coriandrum sativum* L.), and celery (*Apium graveolens* L.) are among the most popular species used worldwide and are aromatic herbaceous plants, members of the family Apiaceae (Umbelliferae) [12,13,14]. In addition, these extracts are used as food ingredients and health promoters [15].

Apium plants belong to the Apiaceae family, and their phytochemical compounds consist of limonene, furanocoumarins, flavonoids, and furocoumarin. These plants are cultivated worldwide for their green leaves, bulbous roots, seeds (fruits), and petioles [15,16,17]. Coriander seeds are among the most important spices in the world and are used in Moroccan cuisine [18]. The essential oil is considered helpful in treating flatulent colic and rheumatism. In addition, coriander is an anti-inflammatory, antidiabetic, antihypertensive, and myorelaxant [19]. *P. crispum* is native to Spain, Italy, Greece, Malta, Algeria, Tunisia, and Morocco. It is a natural additive in food products and a fragrance in cosmetics and perfumes. Anti-microbial, diuretic, and weak antioxidants effects were found in parsley essential oil [20].

In traditional Moroccan pharmacopeia, many genera of this family are used primarily to treat various ailments and symptoms [21], including chills, dyspepsia, poisonous animal bites, ear pain, intestinal pain, cough, mumps, epigastric abdominal pain, allergies, kidney symptoms, infertility, antepartum bleeding, and decreased sexual desire [22,23]. The medicinal plants selected for the present studies are mainly characterized by the presence of high antioxidant components such as phenolic acids and flavonoids [24]. Currently, many researchers studying the antioxidant potential of extracts and essential oils, focusing on their combinations and interactions, have tried to benefit from their synergistic results and improve their effectiveness by lowering the effective dosage [25].

Solvent extraction is the most common approach for isolating active compounds from plant material [26]. Therefore, the type of solvent plays an essential role in the extraction of biologically active compounds of plant origin [27]. This study used ethanol as the extraction solvent due to its low capacity and easy recovery using reduced-pressure distillation [28]. Do et al. confirmed that 100% ethanol exhibited the highest total antioxidant activity, reducing power, and DPPH (2,2-diphenyl-1-picrylhydrazyl) radical scavenging activity [29]. Recent studies by Salih et al. suggested that ethanol was the best solvent for the recovery of TPC and TFC compared to other solvents [30]. Other studies by Sultana et al. have also demonstrated the efficacy of ethanolic solvent in extracting phenolic compounds and antioxidant activity [26]. The same conclusions have been reported by Azza et al., prompting us to select it to design the plant mix [31].

Using a single bioactive compound may increase the risk of disease resistance, especially in long-term treatments. Herbal mixtures are used in various ways to treat specific health problems. The therapeutic efficacy of the combination of medicinal species can be increased with multiple compounds [32]. These suppositions can promote synergy or potentiate a therapeutic effect in the human body, aiming to treat a broader set of conditions or symptoms.

In effect, they are essential for developing new drugs, and their study is of paramount importance today [33,34].

The statistical approach of using designs of experiments (DoEs) is a powerful tool in optimizing mixtures when dealing with multiple components [35]. Designing mixtures based on DoE is a multifactorial approach that relies on well-structured logic and provides faster and more reliable results regarding response optimization [36].

To the best of our knowledge, the use of DoE to study the effect of combinations of the three plants investigated herein has not been previously reported. Our work aims to provide a practical way to design and study an efficient, reproducible, and optimal model with the selected properties to predict an ideal combination characterized by its ability to produce extracts with the highest antioxidant activity and the most significant amounts of TPC from the three plants studied.

## 2. Results and Discussion

### 2.1. Screening Study

**Figure 1:**
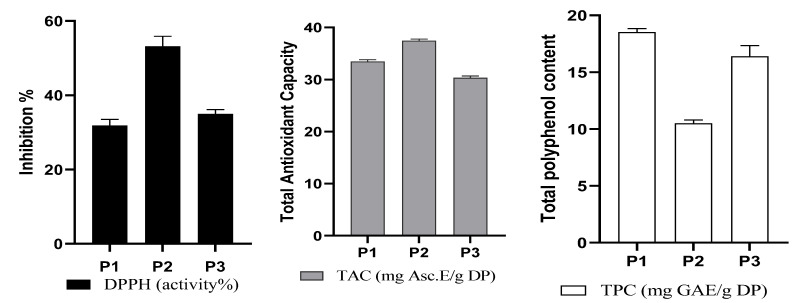
Antioxidant activities (DPPH and TAC) and total polyphenol content (TPC) of the screened plants. Values are means ± SD (n = 3). P1: parsley, P2: coriander, P3: celery.

The primary plant-derived antioxidants are polyphenols, which have greater antioxidant potency than ascorbic acid on a molar basis [37]. Moreover, according to the literature, polyphenols possess many biological activities in vitro [38]. Parsley, coriander, and celery are rich in polyphenols [39]. The results of the total phenolic contents and the antioxidant activity of the three plant extracts are presented in Figure 1.

The values of the total phenolic content in different plant extracts harvested in the Sefrou region in northeastern Morocco using ethanol solvents vary from 10.49 ± 0.30 to 18.52 ± 0.32 mg GAE/g DW; parsley showed the highest amount of polyphenols, followed by celery and then coriander. Further work by Derouich et al. corroborating our results reported TPC results for *C. sativum* L. as 1.372 ± 0.096, for *P. crispum* M. as 2.163 ± 0.104, and for *A. graveolens* L. as 1.739 ± 0.089 g GAE/100 g DW [40]. Additionally, Tang et al. reported high amounts of phenolic compounds in parsley (4.231 g GAE 100 g^−1^) and celery (4.640 g GAE 100 g^−1^) [41]. Different polyphenol contents in coriander seeds were measured (1.555 g GAE 100 g^−1^), and the three plants were also screened using two antioxidant methods [42]. As shown in Figure 1, coriander demonstrated the best total antioxidant activity (37.46 mg AscE/g DW), followed by parsley (33.47 ± 0.29 mg AscE/g DW) and celery (30.32 ± 0.34 mg AscE/g DW). Regarding the DPPH free radical scavenging activity (Figure 1), the hydroethanolic extract of coriander (P2) had the highest DPPH value (53.22%), followed by celery with a value equal to 34.96%, and parsley recorded the lowest trapping potential.

For this screen, the tested samples’ DPPH and TAC radical scavenging activities increased in the order of coriander > celery > parsley.

The results of the recent investigation indicate that the extraction yield and total polyphenol content range from 11% to 23% and from 5.65 to 21.98 mg GAE/g of dried plant, respectively, and DPPH% and TAC activity range from 13.16% to 56.21% and from 12.25 to 75.82 (mg AscE/g), respectively. This result confirms the influence of mixing plants on all the responses studied.

### 2.2. Mixture Design of Experiments

#### 2.2.1. Modeling and Analysis of the Statistical Properties of the Mixture Design

In this study, the Simplex Lattice Mixture scheme was designed to improve the extraction conditions allowing the best recovery of antioxidant compounds, to describe the relationship of the responses of interest (TAC, DPPH, and TPC) as a function of fluctuations in the proportion of the mixture, and to select the optimal ratios of the mixture according to the criteria in Table 1. This approach gathers the maximum information with the fewest analyses.

The evaluation of the quality of the models was tested using the analysis of variance (ANOVA) by Fisher’s test (F-value), the probability value (*p*-value), and the significance of the lack of fit in determining whether the model correctly summarizes the results of the DoE. First, however, the predictive quality of the models was checked using the coefficients of the multilinear regression (R^2^), the predicted coefficient (R^2^_predicted_), and the adjusted coefficient (R^2^_adjusted_). In addition, the plot of average and expected residuals against typical values was used to assess the distribution and normality of the residuals, while the significance of the model was checked using the F-test. After this step, we found that all responses fit better with the cubic model.

#### 2.2.2. Statistical Modeling of the TPC, DPPH, TAC Model

The analysis of variance (ANOVA) results, including regression model terms, R^2^, F-value, and probability values, are illustrated in Table 2. The coefficient of determination (R^2^) value was 0.91. The predicted R^2^ of 0.79 is in reasonable agreement with the adjusted R^2^ of 0.85 for TAC assays, and the R^2^ value is equal to 0.93. The predicted R^2^ of 0.82 follows the adjusted R^2^ of 0.88 for DPPH. In the case of the TPC assay, R^2^, R^2^_adjusted_, and R^2^_Predicted_ were equal to 0.97, 0.95, and 0.93, respectively, and the difference between R^2^_adjusted_ and R^2^_predicted_ is less than 0.2, which indicates that the model adequately represents the actual relationship between the components. Furthermore, the F-values of 54.21, 14.93, and 20.33 (Table 2) for the three activities in the cubic model (TPC, TAC, and DPPH, respectively) imply that the model is significant. There is only a 0.01% probability that an F-value this large could occur due to noise. This coupled with the presentation of small *p*-values (*p*-value < 0.0001) indicates that this model was accurate in predicting the behavior of the mixtures.

The performance of the extract effect is correlated to the coefficient sign [43]. Generally, a negative indication of a coefficient in the fitted model indicates the ability of a variable to decrease the response. In contrast, a positive sign for the coefficient in the model suggests the power of the variable to increase the response [44]. The equation models showed that phenol content and antioxidant activities (TAC and DPPH) were positively and linearly influenced by the mixture AB(A-B); in linear tests, the mixture BC(B-C) gave the lowest coefficient, minor amounts of TPC, and the most insufficient antioxidant activity (DPPH).

The *p*-values and F-values are utilized as evaluation tools, where the smaller the *p*-value and the higher the F-value for each significant coefficient of the cubic model (Table 2), the more important the respective coefficient. In this study, the significant classification of the factors is AB(A-B) > BC > ABC > AB > AC(A-C) > AC > BC(B-C) for the TPC model, AB(A-B) > ABC > BC > AC(A-C) > BC(B-C) > AB > AC for TAC, and AB(A-B) > AB > AC(A-C) >ABC > AC > BC > BC(B-C) for DPPH.

In addition, ternary mixtures with a high proportion ratio of a plant (A) positively influenced the extraction of TPC and antioxidant compounds. The equations obtained from the three models, TPC, TAC, and DPPH, in terms of the natural components, are shown in Table 3. These results indicate that the coefficients determined in the interaction between the three plant extracts revealed the highest synergistic impact.

### 2.3. Diagnostic Plot Analysis

#### 2.3.1. TPC Model

Plant phenolic compounds include phenolics acids, flavonoids, and tannins, which are the most important for dietary applications and the most widely studied [45] due to their ability to eliminate the deleterious effects of oxidation in several ways, including preventing ROS buildup, chelating metal ions [46], inhibiting lipid peroxidation, and enhancing antioxidant enzymes [47]. However, the recovery of phenols is a tedious task [15] which depends on many factors, essentially the nature of the plant [48] and the polarity of the solvent used [26].

Currently, no universal extraction procedure is suitable for extracting all plant phenolic compounds [28]. Thus, it is necessary to establish an optimized process for recovering phenolic compounds from formulations using a combination of three plants.

**Figure 2:**
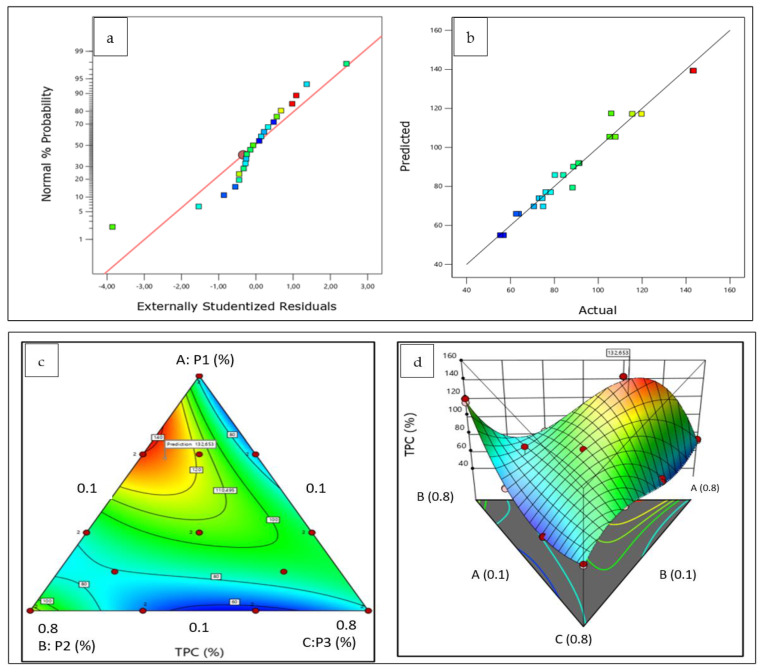
(**a**) Normal probability plot, (**b**) predicted versus actual plot, (**c**) contour plot, and (**d**) 3D surface plot of the effect of the three components on TPC response.

Regarding the validity and accuracy of the model, both plots can confirm the fit of the model, including the standard probability plot (Figure 2a) and the predicted versus actual plot (Figure 2b).

The distribution of the experimental values was close to a straight line, providing a well-fitting model for the determined and anticipated values.

The 3D response surfaces and contour plots acquired for TPC by mixture design as a function of the percentage composition of the three Apiaceae plants’ mixture are illustrated in Figure 2. Each point on the plot represents different proportions of components in the mixture. Three components, *P. crispum* M. (P1), *C. sativum* L. (P2), and *A. graveolens* L. (P3), are included in the 3D surface plots.

The interpretation of the contour lines in Figure 2c,d demonstrates that the extraction of total polyphenols using the herbal mixture with the ratio of 56.67% P1, 33.33% P2, and 10% P3 gave the highest antioxidant activities, which can confirm a synergistic effect between these three herbs, in which the interaction between them demonstrates a better result than their isolated actions.

Moreover, the optimum plant mixture given by the desirability analysis (Table 4) was a ternary mixture that consisted of 61.1% P1, 28.9% P2, and 10% P3.

#### 2.3.2. TAC Model

**Figure 3:**
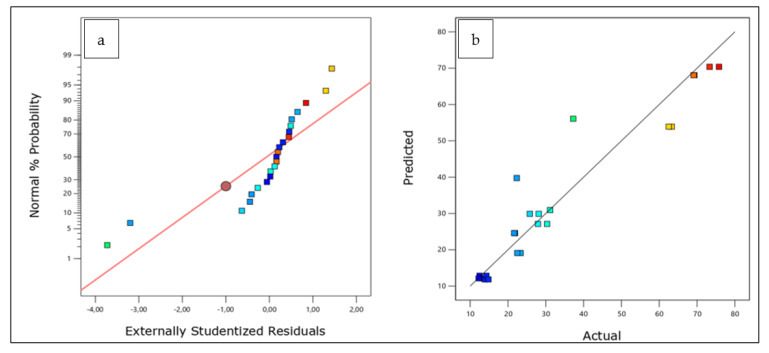

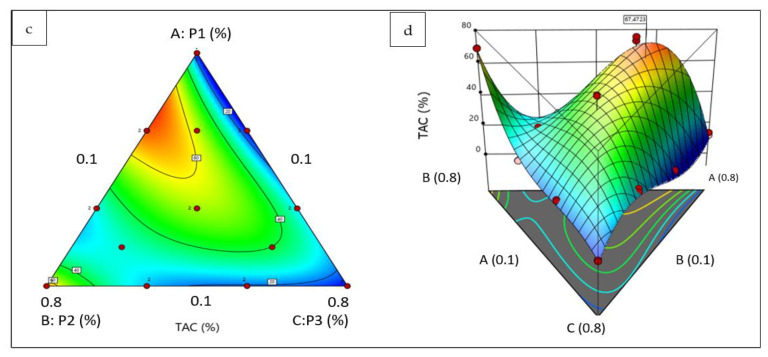
(**a**) Normal probability plot, (**b**) predicted versus actual plot, (**c**) contour plot, and (**d**) 3D surface plot of the effect of the three components on TAC response.

Total antioxidant activity (TAC) represents the ability of a compound to inhibit the oxidative degradation of lipids [49]. Lipid peroxidation involves the oxidative deterioration of lipids with unsaturation used to measure the peroxide level during the initial stage of lipid oxidation. Peroxides are formed during linoleic acid oxidation, which react with Fe^2+^ to form Fe^3+^. These ions form a complex with thiocyanate [50]. The response of the surface plots of the TAC antioxidant assay is shown in Figure 3a,b, illustrating the impact of combining and mixing the three plants. It is seen in Figure 3c,d that when the ratio of plant A (parsley) increased in the mixtures of the three plants, the TAC values increased significantly. On the contrary, when the plant C (celery) ratio increased in the mix, the values of the total antioxidant activity decreased significantly. The simultaneous optimization of the ternary mixture of the three plants led to the optimal formulation based on the functions of desirability, which has the ratios of 0.611, 0.289, and 0.100 (Table 4) for parsley, coriander, and celery, respectively.

The current findings suggest that the formulations have different effects on different antioxidant assays.

#### 2.3.3. DPPH Model

The DPPH assay is used to test the ability of compounds to act as radical scavengers and is frequently used to assess the antioxidant capacity of foods [42,51]. A previous study by Ranneh et al. showed that the herbal mixture significantly affected DPPH scavenging capacity synergistically [52].

The current findings suggest that the formulations have different effects on different antioxidant assays.

**Figure 4:**
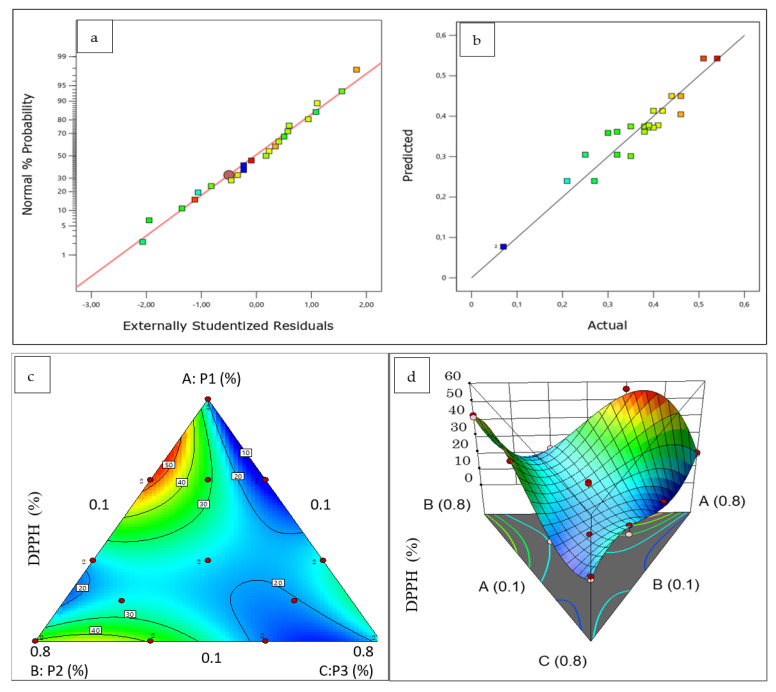
(**a**) Normal probability plot, (**b**) predicted versus actual plot, (**c**) contour plot, and (**d**) 3D surface plot of the effect of the three components on DPPH response.

The effect of the herbal mixture on the antioxidant activities is illustrated in the three-dimensional (3D) surface plot generated from the models (Figure 4). Each point on the graph represents different proportions of components in the mixture. Three components, *P. crispum* M., *C. sativum* L., and *A. graveolens* L., are included in the 3D surface plots; experimental data for the DPPH test, shown in Table 1, demonstrate that the highest DPPH of 56.21% was detected in test 22 under conditions of 56.67% parsley, 33.33% coriander, and 10% celery.

The lowest DPPH (13.65%) was obtained in test 6 under conditions of 56.67% P1, 10% P2, and 33.33% P3. Moreover, the optimal mixture of solvents given by the desirability analysis (Table 4) confirms the same ternary mixture, which consists of 61.1% P1, 28.9% P2, and 10% P3.

The simultaneous optimization of the ternary mixture of the three plants led to the optimal formulation based on the functions of desirability, which has the ratios of 0.611, 0.289, and 0.100 (Table 4) for P1, P2, and P3, respectively.

### 2.4. Numerical Optimization Using Desirability Function

This study aimed to develop a mixture of three plants (parsley, coriander, and celery) according to the criteria described in the material and methods section. Of the two solutions calculated by the software, the value of the function with the highest D was chosen as the optimal solution, which corresponds to the proportions given in Table 1.

The desirability function was optimized to maximize the mixtures’ total phenolics and antioxidant capacity (DPPH and TAC). The results are presented in Table 4. The simultaneous optimization, including all responses, suggested that the ternary mixture, which consists of 61.1% P1, 28.9% P2, and 10% P3, was the most appropriate to obtain the best combination of variables.

## 3. Materials and Methods

### 3.1. Samples

The three plants chosen for this study belong to the Apiaceae family: *Petroselinum crispum* M. (RAB40104), *Coriandrum sativum* L. (RAB76745), and *Apium graveolens* L. (RAB38370), harvested in November 2021. All the plants came from the Sefrou region of Morocco and were identified taxonomically. Their voucher specimen was stored in the Laboratory of Natural Substances, Pharmacology, Environment, Modeling, Health, and Quality of Life (SNAMOPEQ), Faculty of Sciences Dhar El-Mehraz, Sidi Mohamed Ben Abdellah University.

### 3.2. Extraction Procedure

The aerial part of the three plants was used in this study. After drying under shade, plants were powdered and mixed in different proportions. The mixture was then extracted according to the following procedure: a ratio of 1:10 was used to prepare a mixture of the three plants in different proportions. To prepare these mixtures, we added 10 mL of 70% hydroethanolic solution (V/V) to 1 g of each mixture of three plants, and with mechanical agitation, they were macerated for one week in the dark at room temperature. The final extracts obtained were filtered (Whatman, No. 1, Merck KGaA, Darmstadt, Allemagne), and the obtained solutions were concentrated using a rotary evaporator. The extracts were stored at 4 °C until use for the in vitro tests.

### 3.3. Total Phenolic Content (TPC)

A modification of the Folin–Ciocalteu method [31] determined the total phenolic content as follows: 50 µL extract was mixed with 450 μL Folin–Ciocalteu reagent (0.2 N) for 5 min, and then 450 µL sodium carbonate (Na_2_CO_3_) solution (75 g L^−1^) was added. This mixture was incubated at room temperature in dark conditions for 2 h, and the absorbance was read at 760 nm by a UV/visible Jenway 6505 scanning spectrophotometer. The gallic acid solutions underwent the same procedure and were used to plot a calibration curve (standard curve equation, y = 1.0778x + 0.1057, R^2^ = 0.9994) of absorbance against log concentration (mg/mL). The results were calculated as gallic acid equivalent (GAE) per mg of extract. Tests were carried out in triplicate.

### 3.4. Evaluation of the Antioxidant Activity

#### 3.4.1. DPPH Free Radical Scavenging Test

The optimized extracts were tested for the scavenging effect on DPPH (2,2-diphenyl1-picrylhydrazyl) radicals according to the method described by Menezes et al. [53]. A total of 50 μL of the ethanolic extract of the plant mixture was added to 825 μL of ethanolic solution of DPPH (60 μM). After 60 min of incubation, the absorbance was measured at 517 nm using a spectrophotometer. Tests were carried out in triplicate, and the results are given as mean ± SD.

The percentage of inhibition was calculated using the following equation:

(1) $$\mathrm{Inhibition}\;(\%)=\left[\frac{\left(A_{0}-A_{1}\right)}{A_{0}}\right]\times 100$$

where A_0_ is the absorbance of the control (without sample). A_1_ is the absorbance in the presence of the sample.

#### 3.4.2. Total Antioxidant Capacity (TAC) Test

The total antioxidant capacity assay was performed according to El Ghouizi et al. [54] using 1 mL of reagent solution containing 0.6 M of sulfuric acid, 28 mM sodium phosphate, and 4 mM ammonium molybdate with 100 μL of each extract. The solution was then incubated in a water bath at 95 °C for 90 min. The absorbance was then registered at 695 nm against the blank with ethanol in a spectrophotometer (Jasco V-530). TAC determination was carried out in triplicate, and a standard curve (y = 4.2744x + 0.065, R^2^ = 0.9996) of ascorbic acid (Acs. E g^−1^ dry weight) was established.

### 3.5. Experimental Methodology (Mixture Design of Experiment)

The design of the experiment’s approach is a robust tool to understand and optimize the components of a mixture, with a considerable decrease in the number of experiments and a better understanding of the mechanism of the process studied [55]. Herein, the effect of the interaction of *P. crispum* M. (P1), *C. sativum* L. (P2), and *A. graveolens* L. (P3) was studied on three responses: (i) DPPH free radical scavenging activity, (ii) the total antioxidant capacity (TAC), and (iii) the total phenolic content (TPC) was described by the second-order Scheffé model (Equation (2)) [56], so that the predictions of the mixture of plants could be made empirically for each answer and the sum of the proportions of the plants is equal to 1 (Equation (3)), taking into consideration that there is no secondary constraint on the proportion of the components [57].

(2) $$Y=\sum_{i=1}^{p}\beta_{i}xi+\sum \sum_{i<j}^{p}\beta ijxixj+\sum \sum_{i<j}^{p}\delta ijxixj\left(xi-xj\right)+\sum \sum \sum_{i<j<k}\beta ijkxixjxk$$

(3) $$\overset{i=n}{\underset{i=1}{\sum }}xi=1$$

**Figure 5:**
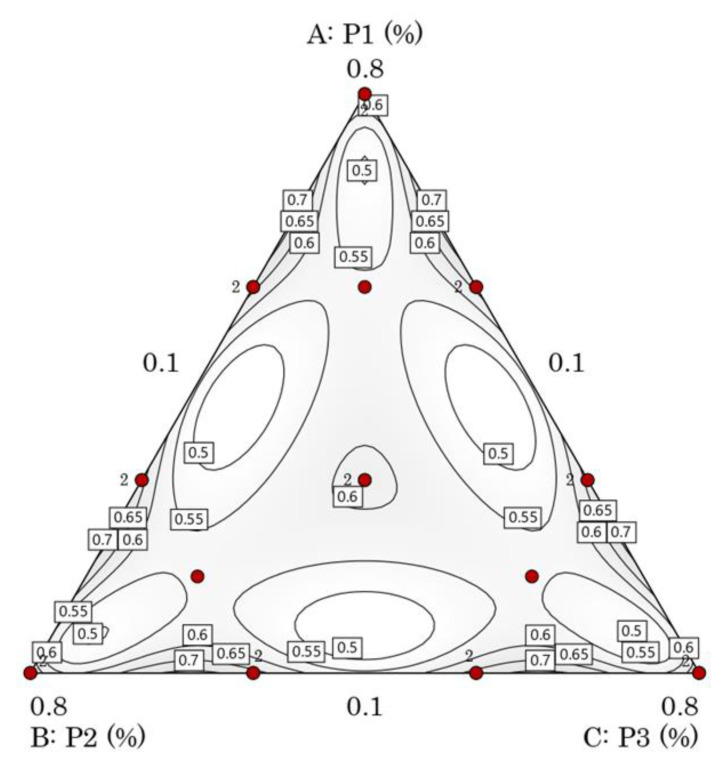
Geometric location of the experimental points of the optimal mixing plan.

In addition, 23 formulations were suggested and conducted in random order using Design-Expert software to account for any randomized hidden effects (Table 5). Table 5 shows the boundaries of the mixture components, and the geometric location of the experimental plan points is illustrated in Figure 5. Once the modeling is complete, the last stage of the study is optimization, which consists of finding the optimal proportions of each plant according to the established constraints. In this study, the numerical optimization method was applied using the desirability function (D) [58], because it allows us to modify the weight and importance of both the components of the mixture and the responses. The limits and constraints selected for optimization using the D function are given in Table 5.

### 3.6. Statistical Analysis

Graph Pad Prism 8.0 was used for the statistical analysis, and comparisons between different samples were performed by ANOVA followed by Tukey’s test.

## 4. Conclusions

The response surface methodology was successfully utilized to optimize the extraction of phenolic antioxidants from three plants of the Apiaceae family. Coriander had the highest phenolic content and thus showed strong antioxidant activities, followed by celery and parsley. The current work is the first report regarding the optimization of phenolic antioxidants in the Apiaceae family and showed that the ternary combination of the three plants was effective, and the best combination was fixed according to the analysis of the software. Our findings enhance the idea of the stimulating effect of the plant towards better antioxidant activities. Our results also prove the possible beneficial use of the optimized formula in the food and pharmaceutical industries. Future study is needed to identify the phytochemical composition to further understand the effects of these extracts.

## Figures and Tables

**Table 1:** Experimental design matrix and mean response values.

Test Runs	Independent Variable				Measured Response		
	Component 1	Component 2	Component 3		Response 1	Response 2	Response 3
	P1 %	P2 %	P3 %	Yield (%)	DPPH (Activity %)	TAC (Mg AscE/g) DW	TPC (Mg GAE/g) DW
1	0.1	0.33	0.57	11.68%	16.67 ± 1.98 ^defghij^	23.33 ± 0.40 ^i^	5.65 ± 0.05 ^bcdefj^
2	0.57	0.33	0.1	20.55%	55.85 ± 0.21 ^a^	73.31 ± 0.11 ^b^	21.95 ± 0.03 ^a^
3	0.1	0.1	0.8	20.99%	23.88 ± 0.32 ^de^	12.25 ± 0.06 ^klm^	7.79 ± 0.05 ^bcd^
4	0.1	0.1	0.8	20.87%	21.54 ± 0.44 ^defg^	13.15 ± 0.06 ^klm^	7.66 ± 0.08 ^bcde^
5	0.33	0.1	0.57	21.64%	26.26 ± 0.93 ^d^	27.92 ± 0.06 ^g^	9.15 ± 0.08 ^bcd^
6	0.57	0.1	0.33	23.24%	13.16 ± 0.14 ^k^	13.90 ± 0.09 ^kl^	7.55 ± 0.08 ^bcde^
7	0.33	0.57	0.1	20.34%	22.63 ± 0.11 ^def^	25.76 ± 0.29 ^h^	8.08 ± 0.08 ^bcd^
8	0.33	0.33	0.33	19.2%	25.32 ± 0.55 ^d^	62.51 ± 0.34 ^d^	10.82 ± 0.05 ^b^
9	0.33	0.57	0.1	19.99%	20.39 ± 0.41 ^defg^	28.12 ± 0.29 ^g^	8.37 ± 0.05 ^bcd^
10	0.8	0.1	0.1	21.68%	18.98 ± 0.48 ^defgh^	12.58 ± 0.23 ^klm^	7.24 ± 0.08 ^bcde^
11	0.22	0.22	0.57	20.45%	20.85 ± 0.34 ^defg^	22.31 ± 0.11 ^ij^	8.92 ± 0.08 ^bcd^
12	0.1	0.8	0.1	19.13%	41.07 ± 0.37 ^b^	69.08 ± 0.46 ^c^	11.51 ± 0.05 ^b^
13	0.8	0.1	0.1	21.88%	18.33 ± 0.35 ^defghi^	14.28 ± 0.06 ^k^	7.49 ± 0.05 ^bcde^
14	0.33	0.33	0.33	20.73%	26.41 ± 0.74 ^d^	63.28 ± 0.29 ^d^	10.53 ± 0.03 ^bc^
15	0.1	0.57	0.33	19.29%	39.26 ± 0.74 ^c^	21.63 ± 0.46 ^ij^	6.28 ± 0.03 ^bcdef^
16	0.57	0.1	0.33	19.87%	13.65 ± 0.53 ^k^	14.83 ± 0.37 ^k^	7.12 ± 0.08 ^bcdef^
17	0.33	0.1	0.57	20.56%	20.60 ± 0.62 ^defg^	30.34 ± 0.03 ^f^	9.17 ± 0.05 ^bcd^
18	0.1	0.57	0.33	19.41%	39.12 ± 0.45 ^c^	21.87 ± 0.40 ^ij^	6.32 ± 0.08 ^bcdef^
19	0.22	0.57	0.22	18.49%	23.54 ± 0.89 ^def^	31.15 ± 0.03 ^f^	8.89 ± 0.08 ^bcd^
20	0.1	0.8	0.1	18.61%	42.38 ± 0.22 ^b^	69.29 ± 0.06 ^c^	11.95 ± 0.03 ^b^
21	0.1	0.33	0.57	20.49%	16.79 ± 0.60 ^defghij^	22.48 ± 0.57 ^ij^	5.57 ± 0.05 ^bcdefg^
22	0.57	0.33	0.1	20.22%	56.21 ± 0.10 ^a^	75.82 ± 0.34 ^a^	21.98 ± 0.03 ^a^
23	0.57	0.22	0.22	20.45%	17.61 ± 0.91 ^defghi^	37.25 ± 0.57 ^e^	5.74 ± 0.08 ^bcdef^

There are significant differences between the mean values (SD, n = 3) that are followed by various letters in the same row (one-way ANOVA; Tukey’s test, *p* < 0.05).

**Table 2:** Coefficients of each model and their level of significance determined by *p*-value.

Variables	TPC			TAC			DPPH		
	Coefficient	*p*-Value	F-Value	Coefficient	*p*-Value	F-Value	Coefficient	*p*-Value	F-Value
Model	12,974.50	<0.0001 *	54.21	9868.98	<0.0001 *	14.93	3149.79	<0.0001 *	18.68
Linear Mixture	2648.64	<0.0001 *	49.80	3307.56	<0.0001 *	22.52	939.76	<0.0001 *	25.08
AB	585.50	0.0004 *	22.02	189.47	0.1322 **	2.58	125.06	0.0227 *	6.68
AC	57.85	0.1641 **	2.18	98.83	0.2669 **	1.35	14.64	0.3927 **	0.7816
BC	2718.60	<0.0001 *	102.22	673.49	0.0097 *	9.17	24.76	0.2710 **	1.32
ABC	850.19	<0.0001 *	31.97	885.63	0.0041 *	12.06	47.52	0.1353 **	2.54
AB(A-B)	5072.34	<0.0001 *	190.73	3819.72	<0.0001 *	52.01	1594.21	<0.0001 *	85.09
AC(A-C)	490.80	0.0009 *	18.45	265.75	0.0795 **	3.62	212.18	0.0051 *	11.33
BC(B-C)	6.31	0.6343 **	0.2373	191.42	0.1304 **	2.61	251.02	0.0029 **	13.40
Residual	345.73			954.73			243.56		
Lack of Fit	311.26	<0.0001 *	30.10	942.78	<0.0001 *	262.96	220.43	<0.0001 *	31.77
R^2^	0.97			0.91			0.93		
R^2^_adjusted_	0.95			0.85			0.88		
R^2^_predicted_	0.93			0.79			0.82		

Level of statistical significance: * Significant, ** not significant; A—*P. crispum* M.; B—*C. sativum* L.; C—*A. graveolens* L.

**Table 3:** The equation of the three models TPC, TAC, and DPPH in terms of the real components.

Response	The Equations
**TPC**	TPC= −20.86 × A + 286.63 × B + 74.07 × C − 72.57 × AB + 4.77 × AC − 611.21 × BC + 1825.67 × ABC + 1335.16 × AB(A-B) − 415.32 × AC(A-C) − 47.1 × BC(B-C)
**TAC**	TAC= −70.38 × A + 236.04 × B − 19.87 × C − 153.96 × AB + 19.65 × AC − 439.2 × BC + 1 863.33 × ABC + 1158.63 × AB(A-B) − 305.61 × AC(A-C) − 259.37 × BC(B-C)
**DPPH**	DPPH = 0.96 × A − 0.34 × B + 0.67 × C − 1.07 × AB − 0.7 × AC + 0.27 × BC + 2.61 × ABC − 7.24 × AB(A-B) + 3.01 × AC(A-C) − 0.19 × BC(B-C)

**Table 4:** Verification experiments under optimal conditions.

Optimal Condition: X_P1_ = 0.611, X_P2_ = 0.289, X_P2_ = 0.100 Desirability: 0.966			
	Predicted	Experimental	Error
TPC (i)	21.22	21.93	0.71 (1.1%) *
TAC (ii)	72.37	72.74	0.37 (0.5%) *
DPPH (iii)	55.05	56.00	0.95 (1.2%) *

* Percent error.

**Table 5:** Components of the mixing plan and its limits.

Component	Name	Units	Type	Coded Low	Coded High
A	P1	%	Mixture	+0 ↔ 0.1	+1 ↔ 0.8
B	P2	%	Mixture	+0 ↔ 0.1	+1 ↔ 0.8
C	P3	%	Mixture	+0 ↔ 0.1	+1 ↔ 0.8

## Data Availability

Not applicable.

## References

[1] Kara M., Assouguem A., Fadili M.E., Benmessaoud S., Alshawwa S.Z., Kamaly O.A., Saghrouchni H., Zerhouni A.R., Bahhou J. (2022). Contribution to the Evaluation of Physicochemical Properties, Total Phenolic Content, Antioxidant Potential, and Antimicrobial Activity of Vinegar Commercialized in Morocco. Molecules.

[2] Wang X.-J., Luo Q., Li T., Meng P.-H., Pu Y.-T., Liu J.-X., Zhang J., Liu H., Tan G.-F., Xiong A.-S. (2022). Origin, Evolution, Breeding, and Omics of Apiaceae: A Family of Vegetables and Medicinal Plants. Hortic. Res..

[3] Tsao R. (2010). Chemistry and Biochemistry of Dietary Polyphenols. Nutrients.

[4] Regassa H., Sourirajan A., Kumar V., Pandey S., Kumar D., Dev K. (2022). A Review of Medicinal Plants of the Himalayas with Anti-Proliferative Activity for the Treatment of Various Cancers. Cancers.

[5] Pandey K.B., Rizvi S.I. (2009). Plant Polyphenols as Dietary Antioxidants in Human Health and Disease. Oxid. Med. Cell. Longev..

[6] Sauceda A.E.Q., Sáyago-Ayerdi S.G., Ayala-Zavala J.F., Wall-Medrano A., de la Rosa L.A., González-Aguilar G.A., Álvarez-Parrilla E., Yahia E.M. (2017). Biological Actions of Phenolic Compounds. Fruit and Vegetable Phytochemicals.

[7] Platzer M., Kiese S., Tybussek T., Herfellner T., Schneider F., Schweiggert-Weisz U., Eisner P. (2022). Radical Scavenging Mechanisms of Phenolic Compounds: A Quantitative Structure-Property Relationship (QSPR) Study. Front. Nutr..

[8] Sies H., Berndt C., Jones D.P. (2017). Oxidative Stress. Annu. Rev. Biochem..

[9] Rahman M.M., Rahaman M.S., Islam M.R., Rahman F., Mithi F.M., Alqahtani T., Almikhlafi M.A., Alghamdi S.Q., Alruwaili A.S., Hossain M.S. (2021). Role of Phenolic Compounds in Human Disease: Current Knowledge and Future Prospects. Molecules.

[10] Gligorijevic N., Radomirovic M., Nedic O., Stojadinovic M., Khulal U., Stanic-Vucinic D., Cirkovic Velickovic T. (2021). Molecular Mechanisms of Possible Action of Phenolic Compounds in COVID-19 Protection and Prevention. Int. J. Mol. Sci..

[11] Thiviya P., Gamage A., Piumali D., Merah O., Madhujith T. (2021). Apiaceae as an Important Source of Antioxidants and Their Applications. Cosmetics.

[12] Lacroix M., Ouattara B. (2000). Combined Industrial Processes with Irradiation to Assure Innocuity and Preservation of Food Products—A Review. Food Res. Int..

[13] Es-safi I., Mechchate H., Amaghnouje A., Kamaly O.M.A., Jawhari F.Z., Imtara H., Grafov A., Bousta D. (2021). The Potential of Parsley Polyphenols and Their Antioxidant Capacity to Help in the Treatment of Depression and Anxiety: An In Vivo Subacute Study. Molecules.

[14] Aćimović M.G., Mérillon J.-M., Ramawat K.G. (2019). Nutraceutical Potential of Apiaceae. Bioactive Molecules in Food.

[15] Majeed M., Hussain A.I., Chatha S.A.S., Khosa M.K.K., Kamal G.M., Kamal M.A., Zhang X., Liu M. (2016). Optimization Protocol for the Extraction of Antioxidant Components from Origanum Vulgare Leaves Using Response Surface Methodology. Saudi J. Biol. Sci..

[16] Cavalcanti V.P., Aazza S., Bertolucci S.K.V., Rocha J.P.M., Coelho A.D., Oliveira A.J.M., Mendes L.C., Pereira M.M.A., Morais L.C., Forim M.R. (2021). Solvent Mixture Optimization in the Extraction of Bioactive Compounds and Antioxidant Activities from Garlic (*Allium sativum* L.). Molecules.

[17] Kooti W., Ali-Akbari S., Asadi-Samani M., Ghadery H., Ashtary-Larky D. (2015). A Review on Medicinal Plant of Apium Graveolens. Future Nat. Prod..

[18] Mandal S., Mandal M. (2015). Coriander (*Coriandrum sativum* L.) Essential Oil: Chemistry and Biological Activity. Asian Pac. J. Trop. Biomed..

[19] Baratta M.T., Dorman H.J.D., Deans S.G., Biondi D.M., Ruberto G. (1998). Chemical Composition, Antimicrobial and Antioxidative Activity of Laurel, Sage, Rosemary, Oregano and Coriander Essential Oils. J. Essent. Oil Res..

[20] Marthe F., Novak J., Blüthner W.-D. (2020). Petroselinum Crispum (Mill.) Nyman (Parsley). Medicinal, Aromatic and Stimulant Plants.

[21] Sousa R.M.O.F., Cunha A.C., Fernandes-Ferreira M. (2021). The Potential of *Apiaceae* Species as Sources of Singular Phytochemicals and Plant-Based Pesticides. Phytochemistry.

[22] Derouich M., Bouhlali E.D.T., Bammou M., Hmidani A., Sellam K., Alem C. (2020). Bioactive Compounds and Antioxidant, Antiperoxidative, and Antihemolytic Properties Investigation of Three *Apiaceae* Species Grown in the Southeast of Morocco. Scientifica.

[23] Çakır D.K., Zannou O., Koca I. (2022). Scopoletin Contents and Antioxidant Properties of Some Edible Plants of Black Sea Regions. Discov. Food.

[24] Lin Y., Tang D., Liu X., Cheng J., Wang X., Guo D., Zou J., Yang H. (2022). Phenolic Profile and Antioxidant Activity of Longan Pulp of Different Cultivars from South China. LWT.

[25] Ouedrhiri W., Mechchate H., Moja S., Baudino S., Saleh A., Al Kamaly O.M., Grafov A., Greche H. (2022). Optimized Antibacterial Effects in a Designed Mixture of Essential Oils of *Myrtus communis*, *Artemisia herba-alba* and *Thymus serpyllum* for Wide Range of Applications. Foods.

[26] Sultana B., Anwar F., Ashraf M. (2009). Effect of Extraction Solvent/Technique on the Antioxidant Activity of Selected Medicinal Plant Extracts. Molecules.

[27] Gupta A., Naraniwal M., Kothari V. (2012). Modern Extraction Methods for Preparation of Bioactive Plant Extracts. Int. J. Appl. Nat. Sci..

[28] Kraemer K., Harwardt A., Bronneberg R., Marquardt W. (2011). Separation of Butanol from Acetone–Butanol–Ethanol Fermentation by a Hybrid Extraction–Distillation Process. Comput. Chem. Eng..

[29] Do Q.D., Angkawijaya A.E., Tran-Nguyen P.L., Huynh L.H., Soetaredjo F.E., Ismadji S., Ju Y.-H. (2014). Effect of Extraction Solvent on Total Phenol Content, Total Flavonoid Content, and Antioxidant Activity of *Limnophila aromatica*. J. Food Drug Anal..

[30] Salih A.M., Al-Qurainy F., Nadeem M., Tarroum M., Khan S., Shaikhaldein H.O., Al-Hashimi A., Alfagham A., Alkahtani J. (2021). Optimization Method for Phenolic Compounds Extraction from Medicinal Plant (*Juniperus procera*) and Phytochemicals Screening. Molecules.

[31] Aazza S. (2021). Application of Multivariate Optimization for Phenolic Compounds and Antioxidants Extraction from Moroccan *Cannabis sativa* Waste. J. Chem..

[32] Achat S. Polyphénols de l’Alimentation: Extraction, Pouvoir Antioxydant et Interactions avec des Ions Métalliques. Ph.D. Thesis.

[33] Youmbai A., Mehellou Z., Boual Z., Gardarin C., Pierre G., Delattre D., Michaud P., Ould El-Hadj M.P. (2021). Characterization and Biological Activities of a Polysaccharidic Extract from *Ferula communis* L. (Apiaceae) Harvested in Sahara. Phytothérapie.

[34] Tunçtürk M., Özgökçe F. (2015). Chemical Composition of Some Apiaceae Plants Commonly Used in Herby Cheese in Eastern Anatolia. Turk. J. Agric. For..

[35] Shamsiev A., Park J., Olawuyi I.F., Odey G., Lee W. (2021). Optimization of Ultrasonic-Assisted Extraction of Polyphenols and Antioxidants from Cumin (*Cuminum cyminum* L.). Korean J. Food Preserv..

[36] Sepahpour S., Selamat J., Abdul Manap M., Khatib A., Abdull Razis A. (2018). Comparative Analysis of Chemical Composition, Antioxidant Activity and Quantitative Characterization of Some Phenolic Compounds in Selected Herbs and Spices in Different Solvent Extraction Systems. Molecules.

[37] Alara O.R., Abdurahman N.H., Ukaegbu C.I. (2021). Extraction of Phenolic Compounds: A Review. Curr. Res. Food Sci..

[38] Ez zoubi Y., Fadil M., Bousta D., El Ouali Lalami A., Lachkar M., Farah A. (2021). Ultrasound-Assisted Extraction of Phenolic Compounds from Moroccan *Lavandula stoechas* L.: Optimization Using Response Surface Methodology. J. Chem..

[39] Mechchate H., Ouedrhiri W., Es-safi I., Amaghnouje A., Jawhari F.z., Bousta D. (2021). Optimization of a New Antihyperglycemic Formulation Using a Mixture of *Linum usitatissimum* L., *Coriandrum sativum* L., and *Olea europaea* Var. *sylvestris* Flavonoids: A Mixture Design Approach. Biologics.

[40] Derouich M., Bouhlali E.D.T., Hmidani A., Bammou M., Bourkhis B., Sellam K., Alem C. (2020). Assessment of Total Polyphenols, Flavonoids and Anti-Inflammatory Potential of Three *Apiaceae* Species Grown in the Southeast of Morocco. Sci. Afr..

[41] Tang D., Chen K., Huang L., Li J. (2017). Pharmacokinetic Properties and Drug Interactions of Apigenin, a Natural Flavone. Expert Opin. Drug Metab. Toxicol..

[42] Yao Y., Sang W., Zhou M., Ren G. (2010). Phenolic Composition and Antioxidant Activities of 11 Celery Cultivars. J. Food Sci..

[43] Majda E., Bouchra L., Faiçal El O., Abdelhak B., Noureddine E. (2020). Application of Response Surface Methodology to Optimize the Extraction of Essential Oil from *Rosmarinus officinalis* Using Microwave-Assisted Hydrodistillation. J. Appl. Pharm. Sci..

[44] Mechchate H., Es-safi I., Amaghnouje A., Boukhira S., Alotaibi A.A., Al-zharani M., Nasr F.A., Noman O.M., Conte R., Amal E.H.E.Y. (2021). Antioxidant, Anti-Inflammatory and Antidiabetic Proprieties of LC-MS/MS Identified Polyphenols from Coriander Seeds. Molecules.

[45] Aquino L.P., Borges S.V., Queiroz F., Antoniassi R., Cirillo M.A. (2011). Extraction of Oil from Pequi Fruit (*Caryocar brasiliense*, Camb.) Using Several Solvents and Their Mixtures. Grasas Aceites.

[46] Dehghani M.H., Hassani A.H., Karri R.R., Younesi B., Shayeghi M., Salari M., Zarei A., Yousefi M., Heidarinejad Z. (2021). Process Optimization and Enhancement of Pesticide Adsorption by Porous Adsorbents by Regression Analysis and Parametric Modelling. Sci. Rep..

[47] Haslam E. (1996). Natural Polyphenols (Vegetable Tannins) as Drugs: Possible Modes of Action. J. Nat. Prod..

[48] Pietta P., Simonetti P., Mauri P. (1998). Antioxidant Activity of Selected Medicinal Plants. J. Agric. Food Chem..

[49] Serpen A., Gökmen V., Fogliano V. (2012). Total Antioxidant Capacities of Raw and Cooked Meats. Meat Sci..

[50] Ayala A., Muñoz M.F., Argüelles S. (2014). Lipid Peroxidation: Production, Metabolism, and Signaling Mechanisms of Malondialdehyde and 4-Hydroxy-2-Nonenal. Oxid. Med. Cell. Longev..

[51] Brand-Williams W., Cuvelier M.E., Berset C. (1995). Use of a Free Radical Method to Evaluate Antioxidant Activity. LWT Food Sci. Technol..

[52] Ranneh Y., Abu Bakar M.F., Ismail N.A., Kormin F., Mohamed M., Md Akim A., Isha A. (2021). Anti-Aging and Antioxidant of Four Traditional Malaysian Plants Using Simplex Centroid Mixture Design Approach. Saudi J. Biol. Sci..

[53] de Menezes B.B., Frescura L.M., Duarte R., Villetti M.A., da Rosa M.B. (2021). A Critical Examination of the DPPH Method: Mistakes and Inconsistencies in Stoichiometry and IC50 Determination by UV–Vis Spectroscopy. Anal. Chim. Acta.

[54] El Ghouizi A., El Menyiy N., Falcão S.I., Vilas-Boas M., Lyoussi B. (2020). Chemical Composition, Antioxidant Activity, and Diuretic Effect of Moroccan Fresh Bee Pollen in Rats. Vet. World.

[55] El Ouadrhiri F., Elyemni M., Lahkimi A., Lhassani A., Chaouch M., Taleb M. (2021). Mesoporous Carbon from Optimized Date Stone Hydrochar by Catalytic Hydrothermal Carbonization Using Response Surface Methodology: Application to Dyes Adsorption. Int. J. Chem. Eng..

[56] Hao H., Zhu X., Zhang X., Zhang C. (2021). R-Optimal Design of the Second-Order Scheffé Mixture Model. Stat. Probabil. Lett..

[57] Aboulghazi A., Bakour M., Fadil M., Lyoussi B. (2022). Simultaneous Optimization of Extraction Yield, Phenolic Compounds and Antioxidant Activity of Moroccan Propolis Extracts: Improvement of Ultrasound-Assisted Technique Using Response Surface Methodology. Processes.

[58] Nunes Filho R.C., Galvan D., Effting L., Terhaag M.M., Yamashita F., de Toledo Benassi M., Spinosa W.A. (2021). Effects of Adding Spices with Antioxidants Compounds in Red Ale Style Craft Beer: A Simplex-Centroid Mixture Design Approach. Food Chem..

